# Biochemical indices, gene expression, and SNPs associated with salinity adaptation in juvenile chum salmon (*Oncorhynchus keta*) as determined by comparative transcriptome analysis

**DOI:** 10.7717/peerj.13585

**Published:** 2022-09-12

**Authors:** Peilun Li, Wei Liu, Wanqiao Lu, Jilong Wang

**Affiliations:** 1 Heilongjiang River Fisheries Research Institute, Chinese Academy of Fishery Sciences, Harbin, China; 2 Key Open Laboratory of Cold Water Fish Germplasm Resources and Breeding of Heilongjiang Province, Heilongjiang River Fisheries Research Institute, Harbin, China

**Keywords:** Chum salmon, Biochemical indices, Transcriptomics, Salinity, Differential expressed genes

## Abstract

Chum salmon (*Oncorhynchus keta*) migrate from freshwater to saltwater, and incur developmental, physiological and molecular adaptations as the salinity changes. The molecular regulation for salinity adaptation in chum salmon is currently not well defined. In this study, 1-g salmon were cultured under 0 (control group, D0), 8‰ (D8), 16‰ (D16), and 24‰ (D24) salinity conditions for 42 days. Na^+^/K^+^-ATPase and Ca^2+^/Mg^2+^-ATPase activities in the gill first increased and then decreased in response to higher salinity environments where D8 exhibited the highest Na^+^/K^+^ATPase and Ca^2+^/Mg^2+^-ATPase activity and D24 exhibited the lowest. Alkaline phosphatase (AKP) activity was elevated in all salinity treatment groups relative to controls, while no significant difference in acid phosphatase (ACP) activity was observed across treatment groups. *De novo* transcriptome sequencing in the D0 and D24 groups using RNA-Seq analysis identified 187,836 unigenes, of which 2,143 were differentially expressed in response to environmental salinity (71 up-regulated and 2,072 down-regulated). A total of 56,020 putative single nucleotide polymorphisms (SNPs) were also identified. The growth, development, osmoregulation and maturation factors of N-methyl-D-aspartate receptors (*nmdas*) expressed in memory formation, as well as *insulin-like growth factor 1* (*igf-1*) and *igf-binding proteins* (*igfbps*) were further investigated using targeted qRT-PCR. The lowest expression of all these genes occurred in the low salinity environments (D8 or D16), while their highest expression occurred in the high salinity environments (D24). These results provide preliminary insight into salinity adaptation in chum salmon and a foundation for the development of marker-assisted breeding for this species.

## Introduction

Salinity, as one of the most important environmental factors in bodies of water, can directly affect the osmotic adjustment, metabolism and energy budget of aquatic organisms, and affect their growth and survival (Silva & Perera, 1976; Galat et al., 1985). The gill plays an important role in osmoregulation and ion regulation under salinity stress. ATPase, including Na^+^/K^+^-ATPase and Ca^2+^/Mg^2+^-ATPase, is an important membrane-bound protease that performs an ion regulation function in gills, providing both a carrier and driving force for ion transport. The liver is an important organ of energy-related metabolic and antioxidative response under salinity stress (Liang et al., 2021; Chang et al., 2021). A variety of physiological stress responses change and produce excessive amounts of reactive oxygen species (ROS) under salinity stress. Fish can use their antioxidant defense systems, such as the superoxide dismutase (SOD), catalase (CAT), glutathione peroxidase (GSH-PX), acid phosphatase (ACP) and alkaline phosphatase (AKP) (Prieto et al., 2007; Zheng et al., 2019), to reduce oxidative stress and protect their tissues from injury (Pungpung, 2007; Tseng & Hwang, 2008).

Salinity-related candidate genes were identified to elucidate the molecular basis and important factors underlying this physiological process. Transcriptome sequencing refers to the technology that detects the transcription reactions of any species under different conditions and provides effective and comprehensive transcriptome information through high-throughput sequencing technology (Ji et al., 2012; Thanh et al., 2014; Nguyen et al., 2016). The transcript expression profile during salinity adaptation is available for many teleost species including: nile tilapia (*Oreochromis niloticus*) (Ronkin et al., 2015), medaka (*Oryzias melastigma*) (Lai et al., 2015), striped catfish (*Pangasianodon hypophthalmus*) (Thanh et al., 2014; Nguyen et al., 2016), hybrid tilapia (*O. mossambicus* female × *O. urolepishornorum* male) (Su et al., 2020) and Asian seabass (*Lates calcarifer*) (Xia et al., 2013).

Two teleost genes, *insulin-like growth factor 1* (*igf-1*) and *igf-binding proteins* (*igfbps*), are important factors in teleost growth, development, osmoregulation and maturation (Wood, Duan & Bern, 2005; Hiroyasu et al., 2008; Reinecke, 2010). These genes are used to determine the growth of biochemical markers in fish (Taniyama et al., 2016). Serum *igf-1* and liver *igfbp-1a* and *igfbp-1b* are correlated with growth rates in chum salmon (*Oncorhynchus keta*) (Taniyama et al., 2016). The exact mechanism of salmon returning behavior is unclear, but it is known that adult salmon utilize olfaction and vision to navigate salinity and rheotaxis changes during homing (Dittman & Quinn, 1996; Putman et al., 2014). Teleosts have a developed olfactory sense that they use for finding food and partners and for communicating with others (Hara, 2010; Sorensen et al., 1995). N-methyl-D-aspartate receptors (*nmdas*) are glutamate receptors expressed in memory formation (Sison & Gerlain, 2011), which are composed of *nmda1* and four *nmda2s* (*nmda2a-d*) (Cox, Kucenas & Voigt, 2005; Kinoshita et al., 2005), and have been the focus of many olfactory memory studies.

The chum salmon is a long-distance migratory fish and one of the six Pacific salmon species (Li et al., 2017). They can be found in the Heilongjiang River, Wusuli River, Suifen Current and Tumen River in China. Chum salmon hatch in a freshwater environment weighing approximately 1 g. They then grow, gradually adapt and migrate to seawater (Taniyama et al., 2016). The spawning groups migrate to the river in autumn, reproduce only once, and then die. Previous chum salmon research has mostly focused on growth aspects, olfactory hormones (Ueda et al., 2016), environmental DNA (Minegishi et al., 2019) and gene expression in different tissues (Kim et al., 2015; Taniyama et al., 2016; Takashi & Hideaki, 2019).

In this study, chum salmon were placed in different salinities for 42 days. The ATPase and antioxidant enzymes were investigated and the whole fish were sequenced by RNA-seq. The transcriptomic data was compared and analyzed to identify salinity-related genes and pathways, and different SNPs positions were found. The results of this study will help to illustrate the mechanism of euryhaline fish adaptation under different salinity environments.

## Materials and Methods

### Animals and disposal

The fish used in this study came from the Tangyuan breeding base of the Heilongjiang River Fisheries Research Institute, Chinese Academy of Fishery Sciences. The chum salmon, weighing 1.01 ± 0.135 g, were bred in fully automatic temperature-controlled aquariums (80 cm × 60 cm × 50 cm) with a portable filter system in the aquaculture workshop of the Heilongjiang Fisheries Research Institute. Our study was designed with four groups: 0 saline (the control group, D0), 8‰ saline (D8), 16‰ saline (D16), and 24‰ saline (D24), and three replicates were set up in each group. The water used in the experiment was prepared with underground water and sea salt (Haike Ocean, Qingdao, China), and the salinity was calibrated using a salinity meter (HQ4300; HACH, Loveland, CO, USA). Salinity domestication was carried out in a step-by-step method. Salinity was increased 4‰ every day in each test group until it reached the target salinity. There were 360 healthy fish included in this experiment, randomly assigned to different groups. The experimental fish were fed twice a day using special pellet feed for salmon (Salmofood, Los Lagos, Chile). The water temperature was controlled at 14 ± 0.5 °C, pH 8.0 ± 0.5, and the dissolved oxygen content in water was above 9 mg/L. The water was changed every 3 days, and the test period was 42 days. The sample fish were not fed for 24 h before sampling and then anesthetized using MS-222 at a concentration of 90 mg/L. In this study, 30 fish were randomly collected from each group. The gills and livers of some fish were dissected immediately to determine enzyme activity. The remaining fish were immersed in liquid nitrogen immediately after removing their tails for transcriptome sequencing and qRT-PCR. All samples were then stored at −80 °C for the next experiment. After the completion of all experiments, the surviving test fish were transported to Tangwang River for release. All animal experiments were conducted in accordance with the guidelines and approval of the Animal Research and Ethics Committees of Heilongjiang River Fisheries Research Institute, and the approval number was HSY20180311.

### Enzyme activity assays

The Na^+^/K^+^-ATPase and Ca^2+^/Mg^2+^-ATPase activity levels in the gills were measured according to the methods outlined by Quabius, Balm & Bonga (1997) and Zhou, Liang & Zhang (2012). The activity levels of superoxide dismutase (SOD), glutathione peroxidase (GSH-PX), catalase (CAT), acid phosphatase (ACP) and alkaline phosphatase (AKP) in the liver were measured according to various enzyme assay kits (Nanjing Jiancheng Bioengineering Institute, Nanjing, China). The analysis of covariance (ANCOVA) was used to test the experimental data using the SPASS 19.0 software, and a *P*-value < 0.05 indicated a significant difference.

### RNA extraction, transcriptome library preparation and lllumina sequencing

The total RNA in the whole fish without the tail was extracted from the chum salmon using TRIzol® Reagent (TransGen Biotech, Beijing, China). The RNA concentration and quality was analyzed using a Nano Drop 2000 spectrophotometer (Thermo Scientific, Waltham, MA, USA), and the integrity of the total RNA were confirmed with 1% agrose gel electrophoresis and the Bioanalyzer 2100 (Agilent technologies, Santa Clara, CA, USA), respectively. The RNA of an OD260/280 ≥1.8 and a concentration ≥100 ng/μL was selected for the experiments. The cDNA was synthesized using cDNA Synthesis SuperMix according to the manufacturer’s instructions (TransGen Biotech, Beijing, China) for qRT-PCR.

A total of 1 μg RNA per sample was used for the RNA sample preparations. Sequencing libraries were generated using the NEBNext®Ultra™ RNA Library Prep Kit for Illumina® (NEB, Ipswich, MA, USA) following the manufacturer’s recommendations and index codes were added to attribute sequences to each sample. PCR products were purified (AMPure XP system) and the library quality was assessed on the Agilent Bioanalyzer 2100 system.

### Basic analysis of sequencing data, functional annotation and expression analyses

Clean reads were obtained by removing low-quality reads and reads containing adapter sequences or poly-N from the raw reads, Q30. The GC-content and sequence duplication level of the clean data were then calculated, and the clean reads were analyzed in the D0 and D24 groups using Trinity with default parameters. RNASeqPower (https://doi.org/doi:10.18129/B9.bioc.RNASeqPower) was used to make the power analysis calculation and edgeR (Robinson, McCarthy & Smyth, 2010) was used to find the read depth in this study.

The NCBI non-redundant protein sequences (nr) and non-redundant nucleotide sequences (nt) were searched to annotate the assembled unigenes of chum salmon using local the BLASTX and BLASTN programs with a 1 × 10^−5^ E-value (Altschul et al., 1997). All unigenes were further annotated on the protein family (pfam), Clusters of Orthologous Groups of proteins (KOG/COG), Swiss-Prot (A manually annotated and reviewed protein sequence database), Gene Ontology (GO), EC (Enzyme Code) and Kyoto Encyclopedia of Genes and Genomes (KEGG) (https://www.genome.jp/kegg/kegg3.html) databases using Blast2GO (Conesa et al., 2005).

The expression levels of different genes were calculated with FPKM (fragments per kilobase per million fragments mapped) (Mortazavi et al., 2008) and a differential expression analysis of the D0 and D24 groups was performed by the DESeq2 R package with the false discovery rate correction set at FDR < 0.05 and the absolute value of |log_2_FC| (fold change) > 2 as the threshold to judge significance (Benjamini & Yekutieli, 2001). The Venny online software (http://bioinfogp.cnb.csic.es/tools/venny/) was used to combine the analyses of differentially expressed genes. A Gene Set Enrichment Analysis (GSEA) was also used to detect the expression changes of the whole gene set.

### Growth-related and memory-related gene validation

The growth-related genes, *igf-1*, *igfbp-1a* and the *igfbp-1b* primers used for qRT-PCR were the same as those used by Kawaguchi et al. (2013). The gene-specific qRT-PCR primers of memory-related genes *nmda1*, *nmda2b* and *nmda2c* and *β-actin* (AB032464) were used as outlined by Kim et al. (2015) and shown in Table 1. The qRT-PCR was performed using TransStart Top Green qPCR SuperMix (TransGen Biotech, Beijing, China) according to the manufacturer’s instructions in an ABI 7300 Real-Time PCR System (Applied Biosystems, Foster City, CA, USA). One denaturation cycle was performed at 95 °C for 5 min, and the qRT-PCR cycle was as follows: 95 °C/30 s, followed by 40 cycles of 95 °C for 5 s, and 60 °C (*igf-1*, *igfbp-1a* and *igfbp-1*) or 55 °C (*nmda1*, *nmda2b* and *nmda2c*) for 30 s. The gene expression levels of *β-actin* and the control group genes were used as the internal control and the reference, respectively. All samples were run in triplicate. The analysis of covariance (ANCOVA) was used to test the experimental data using the SPASS 19.0 software, and the averages of the three relative quantities of the biological replications were subjected to a two-tailed Student’s *t* test with a 95% confidence level (*P* < 0.05) to determine the gene expression significance.

**Table 1:** The primer sequences of growth-related and memory-related genes for qRT-PCR.

Annotation	Forward primer (5′-3′)	Reverse primer (5′-3′)
*igf-1*	TCTCCAAAACGAGCCTGCG	CACAGCACATCGCACTCTTGA
*igfbp-1a*	AAGGAGCGGCGGACAATG	CTGTGGCCGTGGAGATAGAG
*igfbp-1b*	GACAAGGGACAAGAGGTAGTAGAAT	GCTCTCCTGATTCCCCTCAT
*nmda1*	CAGGCGAACCAGATATACG	AGGATGACTCACGAGGATG
*nmda2b*	CATCCTCATGCTGTTCGG	TGTAGAAGACACCTGCCAT
*nmda2c*	GGAAGCACAGAGAGGAACA	GCACAGCAGCGTCATAGA

### Single-nucleotide polymorphism (SNPs) analysis

The chum salmon SNPs were found in the transcriptome sequences. SAMtools 0.1.19 (Li et al., 2009) was used to sort, remove duplicated reads and merge the bam alignment results of each sample. GATK 2.8-1 (Mckenna et al., 2010) was used to perform SNPs. Raw vcf files were filtered with the GATK standard filter method and other parameters (cluster Window Size: 10; MQ0 ≥= 4 and (MQ0/(1.0 * DP)) > 0.1; QUAL < 10; QUAL < 30.0 or QD < 5.0 or HRun > 5), and only SNPs with a distance >5 were retained.

## Results

### Enzyme activity

**Figure 1:**
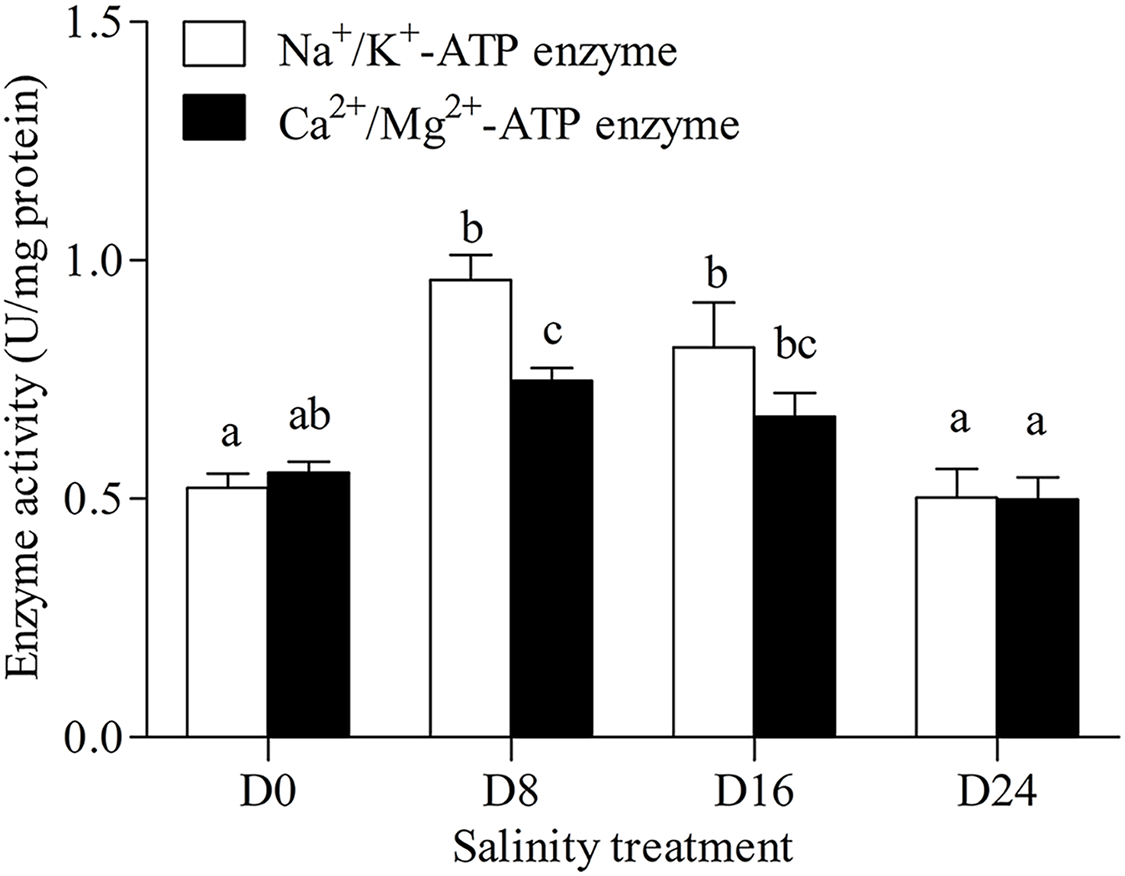
Activity assay of Na^+^/K^+^-ATPase and Ca^2+^/Mg^2+^-ATPase in chum salmon gills under different salinities. The units for the activity assay are U/mg protein, and the values are the means ± SD, *n* = 6. Different letters denote significant differences (*P* < 0.05) between salinity groups.

Na^+^/K^+^-ATPase and Ca^2+^/Mg^2+^-ATPase activities in the gills of the chum salmon tended to first increase and then decrease as salinity increased; the D8 and D24 salinity groups had the highest and lowest activity, respectively (Fig. 1).

**Figure 2:**
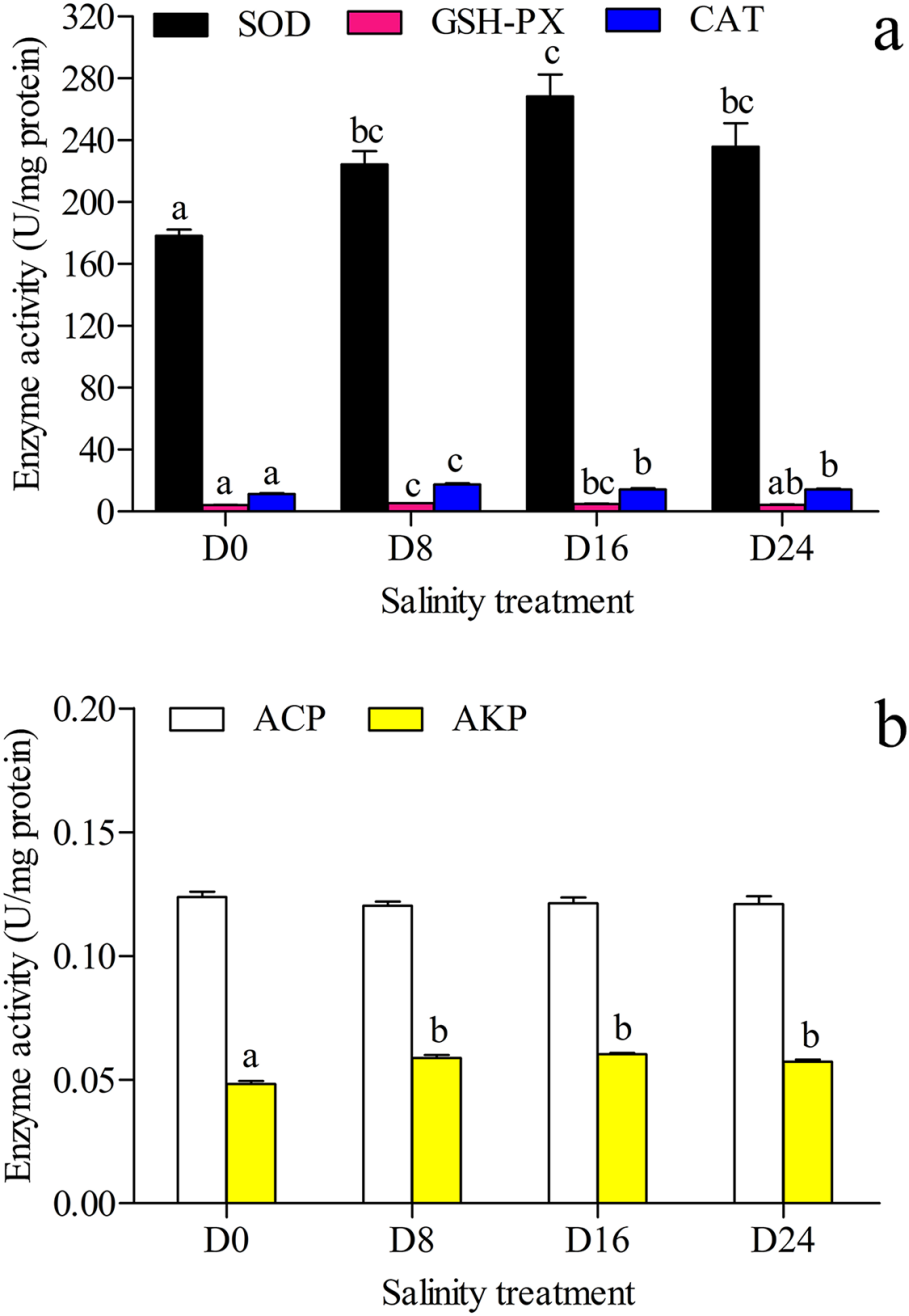
Activity assay of antioxidases and hydrolases. (A) The superoxide dismutase (SOD), glutathione peroxidase (GSH–PX) and catalase (CAT) activity in chum salmon liver under different salinities. (B) The acid phosphatase (ACP) and alkaline phosphatase (AKP) activity in chum salmon liver under different salinities. The units for the activity assay are U/mg protein, and the values are the mean ± SD, *n* = 6. Different letters denote significant differences (*P* < 0.05) between salinity groups.

The antioxidant and hydrolase activities of SOD, GSH–PX, CAT, ACP and AKP in the liver were investigated, and the results are shown in Figs. 2A and 2B. The highest SOD activity and AKP activity were found in the D16 group, and the lowest SOD activity and AKP activity were seen in the D0 group; the highest GSH–PX activity and CAT activity were seen in the D8 group, and the lowest GSH–PX activity and CAT activity were found in the D0 group. The AKP activity in the D0 group was significantly lower than that in the other groups, and there was no significant difference in ACP activity between these groups.

### Transcriptome analysis

Ambiguous nucleotides, low-quality and short sequences were removed from the results of the transcriptome analysis (Table S1). There was an average length of 606 bp in 187,836 unigenes, and N50 lengths of 998 bp were found. Approximately 50% of the unigenes ranged from 200 to 500 bp (Fig. S1), 25,859 (13.77%) unigenes exceeded 1,000 bp, and 11,195 (5.96%) exceeded 2,000 bp. The transcriptome functional annotation was searched using the NCBI nr, Swiss-Prot, KEGG, COG, KOG, GO and Pfam databases. The raw data has been submitted to NCBI (https://www.ncbi.nlm.nih.gov/), and freely downloaded from the SRA database with the name: “Transcriptome of juvenile chum salmon in different salinity” and the accession number was PRJNA778360. A power analysis was calculated in the D0 and D24 groups (Figs. S2 and S3).

**Figure 3:**
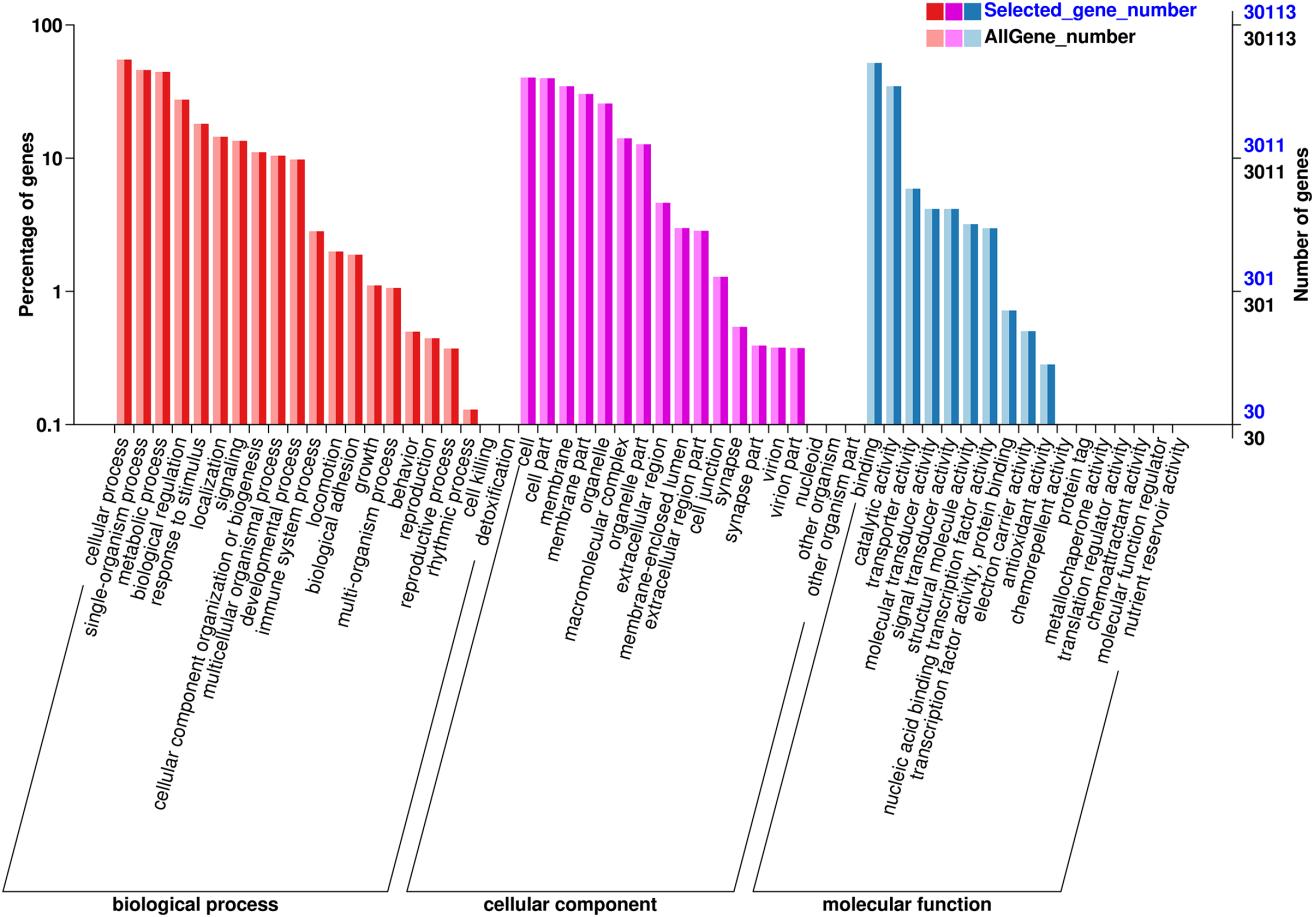
GO annotation of the chum salmon transcriptome. Unigenes were annotated by Gene Ontology (GO) terms that belong to three categories: biological process, cellular component and molecular function.

The functions of the unigenes were predicted and classified against the GO database, which were annotated in three major GO categories: 68,326 (41.36%) genes in the biological process (BP) category, 63,791 (38.61%) genes the cell component (CC) category, and 33,642 (20.03%) genes in the molecular function (MF) category (Fig. 3). In the BP category, most unigenes were related to cellular process (13,936 unigenes, GO:0009987), metabolic process (10,787 unigenes, GO:0008152), and the single-organism process (11,165 unigenes, GO:0044699). The 12,119 unigenes (GO:0005623) in the CC categories were involved in the cell, cell part (11,981 unigenes, GO:0044464), and the membrane (10,451 unigenes, GO:0016020). In the MF category, the unigenes were predicted for binding (15,556 unigenes, GO:0005488), catalytic activity (104,233 unigenes, GO:0003824), and transporter activity (1,775 unigenes, GO:0005215).

**Figure 4:**
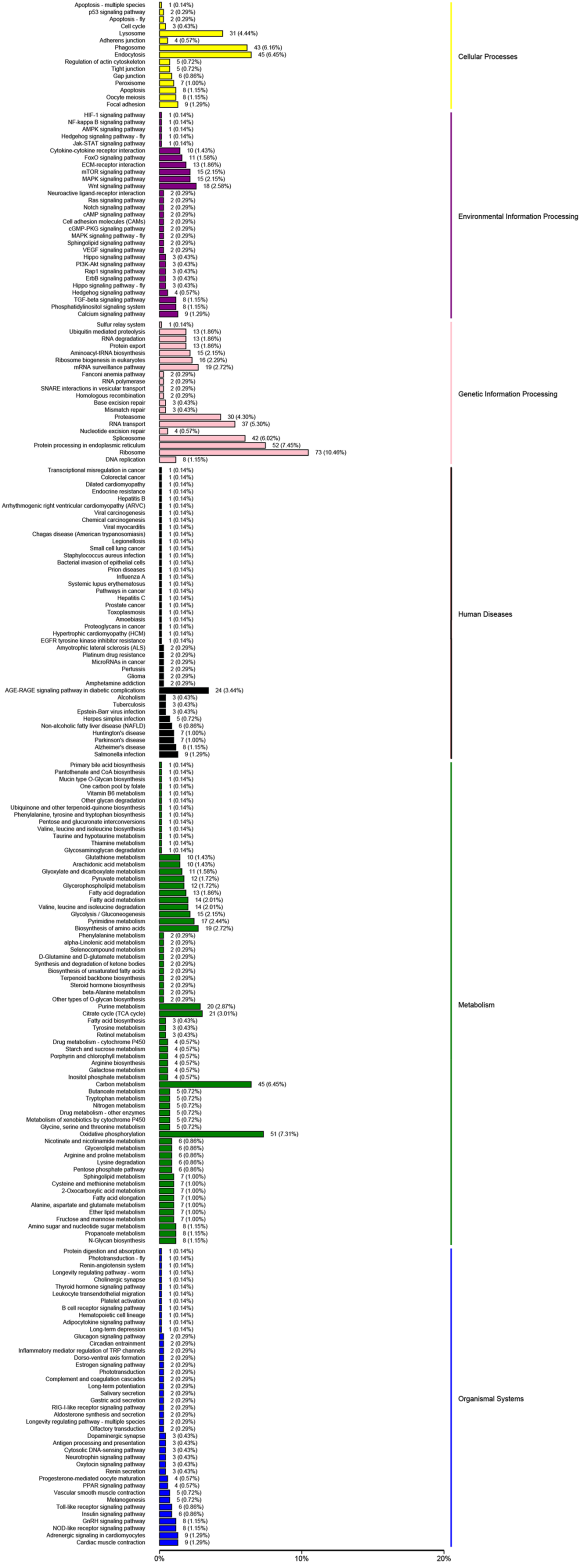
KEGG pathways were grouped into six main clusters: cellular processes, environmental information processing, genetic information processing, human diseases, metabolism and organismal systems.

The KEGG analysis could help to identify the key pathways associated with salinity changing. In this study, 14,591 unigenes were clustered in six major categories: cellular processes, environmental information processing, genetic information processing, human diseases, metabolic and organismal systems (Fig. 4). For KEGG groups, the metabolic pathway annotations were “oxidative phosphorylation” (407 unigenes, KO00190) and the “MAPK signaling pathway” (724 unigenes, KO04020); the “mTOR signaling pathway” (486 unigenes, KO04150) was associated with environmental information. The “Insulin signaling pathway” was the most enriched group in the organismal system (471 unigenes, KO04910). In the human disease group, “Herpes simplex infection” (491 unigenes, KO05618) was enriched in a high proportion of the unigenes.

### Different expression genes analysis

**Figure 5:**
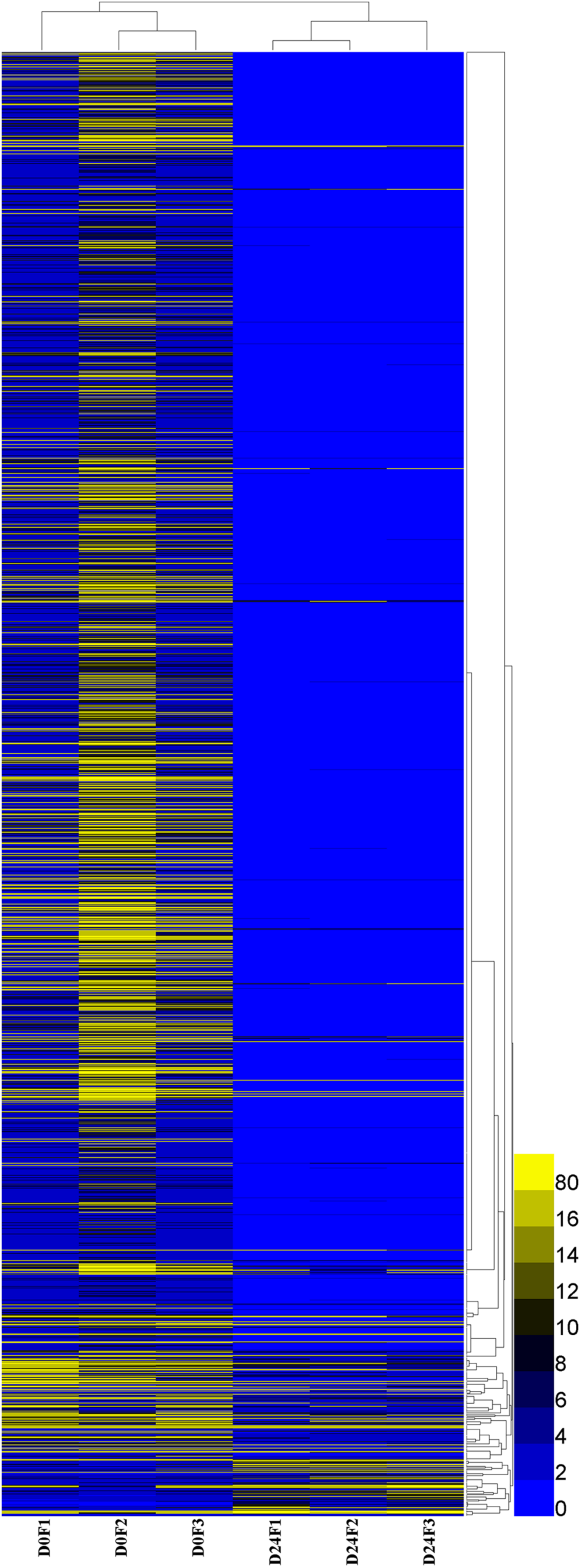
Differential gene expression pattern analysis. FPKM is the number of fragments per kilobase length of a certain gene per million fragments. D0 was the control group, D24 was the salinity group.

A total of 23,528 genes co-expressed in the D0 and D24 groups (Fig. S4) and the FPKM values of genes in every group were calculated. A total of 2,143 genes were significantly and differentially expressed in the chum salmon transcriptome (Fig. 5), and 71 up-regulated genes and 2,072 down-regulated genes were identified.

The DEGs GO enrichment analysis was selected to annotate the differentially expressed genes. These results showed that differentially expressed genes were divided into three categories: molecular function, biological process and cellular component. The salinity-related genes were further selected in GO function terms, which showed that the 56 salinity genes were only found in biological processes, and associated with the following responses: hyperosmotic salinity response (32 genes), cellular hypotonic salinity response (six genes) and hypotonic salinity response (18 genes); these genes were all down-regulated in our analysis.

We used the DEGs KEGG enrichment analysis to annotate the differentially expressed genes. The up-regulated genes were found in the MAPK signaling pathway, the calcium signaling pathway, the environmental information processing cluster and oxidative phosphorylation in the metabolism cluster (Fig. S5A). The down-regulated genes were expressed mainly in the Wnt signaling pathway, the MAPK signaling pathway and the mTOR signaling pathway in the environmental information processing cluster, the oxidative phosphorylation in the metabolism cluster, the AGE-RAGE signaling pathway in diabetic complications and Salmonella infection in the human disease cluster (Fig. S5B).

### The expression of growth-related genes and memory-related genes

**Figure 6:**
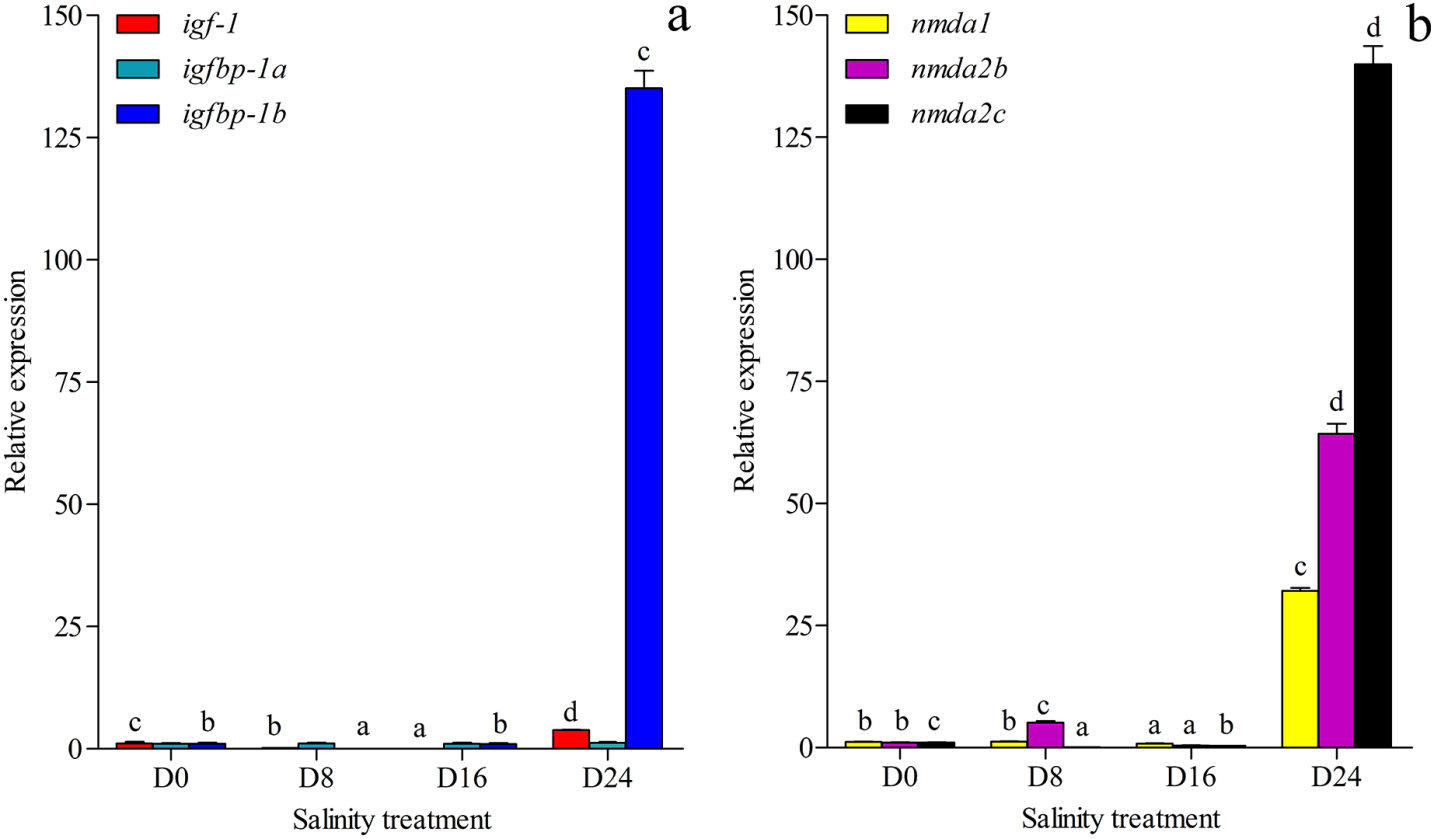
Gene expression. (A) Expression of *igf-1*, *igfbp-1a* and *igfbp-1b* in chum salmon under different salinities; (B) Expression of *nmda1*, *nmda2b*, *nmda2c* in chum salmon under different salinities. The whole fish were selected for qRT-PCR. Each sample was tested in triplicate. qRT-PCR fold changes are relative to control samples (D0 group) and are normalized by changes in β-actin values. The averages of the three relative quantities of the biological replications were subjected to two-tailed Student’s *t* test with a 95% confidence level (*P* < 0.05) to determine the gene expression significance, and the different letters denote significant differences (*P* < 0.05) between salinity groups.

The growth-related genes and memory-related gene expression was investigated and annotated to the transcriptome database in this study (Figs. 6A and 6B). The expression of growth-related gene *igfbp-1a* (ON804215) was less affected by changes in salinity levels, but the expression levels of the *igf-1* (ON804216) and *igfbp-1b* (ON804217) genes were more affected by salinity changes. The gene expression of *igf-1* was lowest in the D16 group and highest in the D24 group. The gene expression of *igfbp-1b* was lowest in the D8 group and highest in the D24 group. The expression of memory-related genes was more strongly affected by changes in salinity: the lowest expression of *nmda1* (ON804218) and *nmda2b* (ON804219) were in the D16 group, and the expression of *nmda2c* (ON804220) was lowest in the D8 group, and these genes showed the highest expression in the D24 group. The expression trend of these genes was similar to the transcriptome analysis results. Overall, the results showed that the relative expression of growth-related genes and memory-related genes was lowest in the D8 or D16 group, and highest in the D24 group.

### SNPs

**Figure 7:**
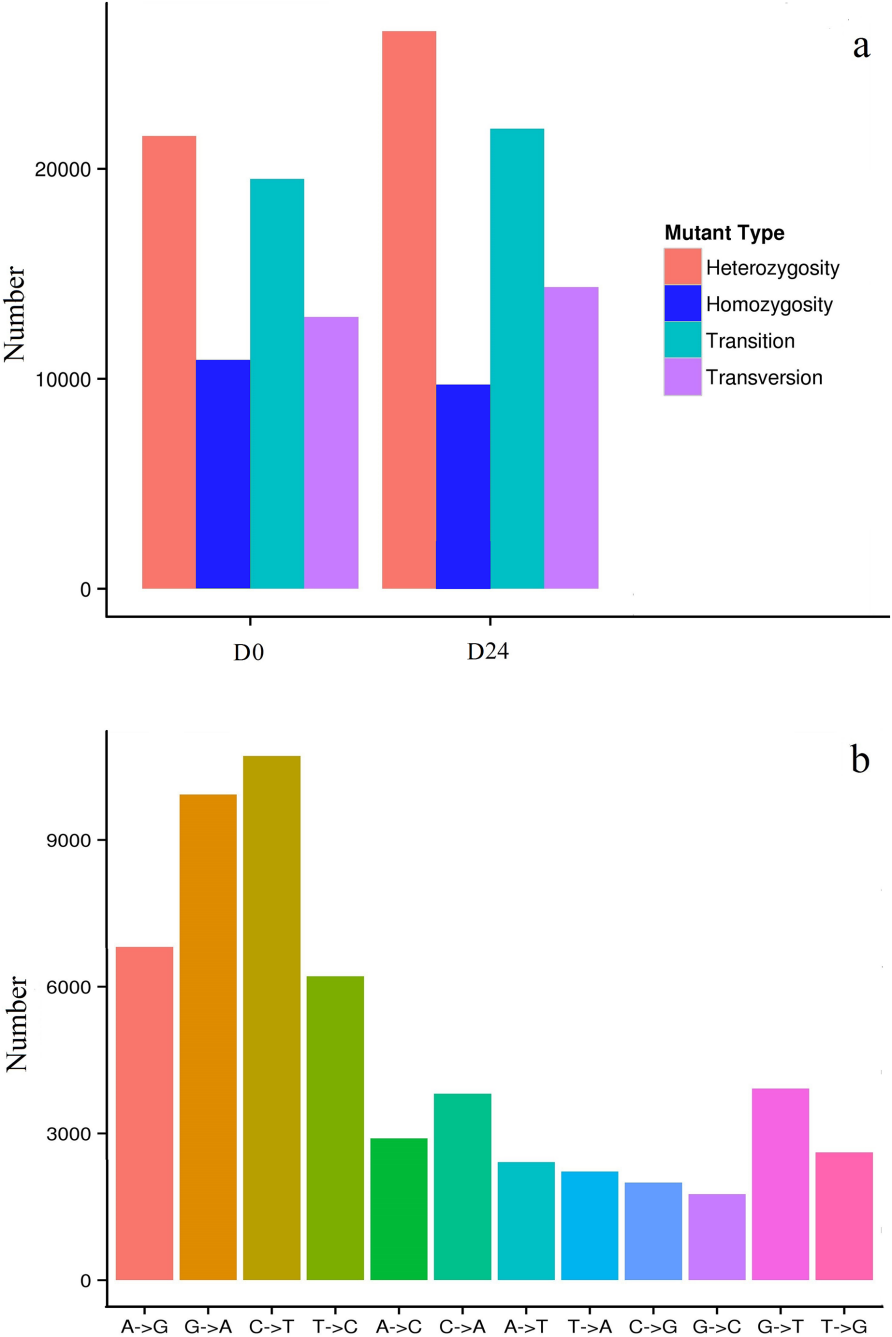
Distribution of SNPs in the chum salmon assembled transcriptome.

In this study, 32,468 SNPs (19,523 transitions and 12,945 transversions) in the D0 group and 36,289 SNPs (21,920 transitions and 14,369 transversions) in the D24 group (Fig. 7A) were identified. More than 50,000 SNPs were identified, some as heterozygotes and some as homozygotes for different nucleotides in the D0 and D24 groups (Table S2). Among the SNPs, the most abundant types were the AG/GA and CT/TC types, while the GC/CG types were least abundant compared to the other types (Fig. 7B) and there were 56,020 SNPs positioned that differed between the two groups.

## Discussion

### Effects of salinity on the enzyme activity in chum salmon

As chum salmon migrate from freshwater to saltwater, they change in response to the changes in salinity. In our study, the salinity levels ranged from 0‰ to 24‰ according to the salinity levels of the migratory stages of chum salmon. Some marine fish spend some metabolic energy in the osmotic-regulatory process in a salinity variation environment (Marais, 1978; Moser & Miller, 1994). The gill has many important functions in addition to being the respiratory organ of teleost fish, including: regulating the osmotic pressure balance of the body, regulating body fluid pH, ion transport and excretion of ammonia nitrogen (Ern & Esbaugh, 2018). Euryhaline fish have a high salinity survival range, and the organizational structure and physiological function of gills could have adaptive changes during the migration from freshwater to saltwater (Shui et al., 2018). This may result in significant changes in ATPase activity related to osmotic regulation due to salinity concentration and exposure time (Monserrat et al., 2007). In Nile tilapia, Mg^2+^-ATPase activity showed fluctuation trends, Na^+^/K^+^-ATPase activity increased and Ca^2+^-ATPase activity decreased in different levels of salinity (Kulac, Atli & Canli, 2013). In this study, Na^+^/K^+^-ATPase and Ca^2+^/Mg^2+^-ATPase activities showed a trend of first increasing and then decreasing with the increase of salinity, with the D8 group showing the highest activity in this study. ATPase activity in the gills of euryhaline teleost fish varied with different concentrations of ions affinities in the salinity variation environment and the activity levels of antioxidant enzymes changed with external factors, such as pH and salinity levels (Hegazi, Attia & Ashour, 2010; Xu et al., 2014). These results indicate that gill ATPase activity could increase with proper salinity in aquaculture water, but could also decrease if salinity levels increased beyond a certain range.

The oxidative status and cellular production of ROS are influenced by different environments (Chang et al., 2021). The liver is an important organ involved in the antioxidative response (Martínez-Álvarez, Morales & Sanz, 2005) and the antioxidant enzymes of SOD, CAT and GSH-PX in the liver can eliminate ROS in the antioxidative response. In fact, the physiological processes changed for maintaining balance under the pressure and energy of the osmotic regulation: ATPase enzyme activity increased to transport ions under osmotic pressure, digestive enzyme activity increased for food digestion and absorption, and ROS increased production to attenuate oxidative stress (Kulac, Atli & Canli, 2013; Martínez-Álvarez, Morales & Sanz, 2005; Liu et al., 2018). In this study, SOD, GSH-PX, and CAT significantly varied in the different salinity groups. These results could indicate that ROS scavengers could increase in a low salinity environment, and decrease in a higher salinity environment. ACP and AKP are part of the non-specific immune system and promote the hydrolysis of phosphate into inorganic phosphoric acid and the production of ATP; AKP is also involved in nutrient absorption and protein synthesis (Wu et al., 2019). In this study, AKP activity in the salinity groups was significantly higher than in the control group, but there was no significant difference in activity between the salinity groups. This result indicates that AKP could be a key enzyme of ATP production in chum salmon during salinity changes.

### Transcriptome analysis of chum salmon under salinity stress

Chum salmon have an excellent osmotic plasticity in response to hyperosmotic and hypoosmotic environments. In previous studies, the gill osmotic regulatory proteins of chum salmon were selected for a comparative transcriptome, and the fish were exposed to a salinated environment for 1 day (Lee, Lee & Kim, 2020). The olfactory transcriptome was also analyzed in homing chum salmon (Palstra et al., 2015). In our study, chum salmon were placed in different salinities for 42 days, and the whole fish without tail were analyzed with RNA-seq in the D0 and D24 groups. The different unigenes of whole chum salmon without tail were identified and annotated, 2,143 genes were significantly and differentially transcribed. The differentially expressed genes were annotated using a GO enrichment analysis, and the results showed that these genes were related to the hyperosmotic and hypotonic salinity responses. These results suggest that the osmosis-related genes were regulated to adapt to the salinity change.

Osmotic pressure regulation uses several ion transport channels, which require a large amount of energy (Tseng & Hwang, 2008). In this study, most DEGs were annotated to the energy metabolism pathway of oxidative phosphorylation. Oxidative phosphorylation has been shown to be relevant to osmoregulation in *Acipenser baerri* in a study which found that there were 51 DEGs associated with this pathway (Guo et al., 2018). Chum salmon could also provide the energy needed to adapt to salinity changes using oxidative phosphorylation. In this study, there were 96 differential genes in the environmental process, mainly in the MAPK signaling pathway and the mTOR signaling pathway. The MAPK (mitogen-activated protein kinase) signaling pathway is important in muscle cell proliferation and differentiation (Jones et al., 2001; Ren, Accili & Semenza, 2010), and the mTOR (mammalian target of rapamycin) signaling pathway mediates signaling in response to nutrient availability, cell energy, mitogenic signals and various types of stressors (Campos et al., 2014). The innate immune response is important in fish (Watts, Munday & Burke, 2011), and a large number of immune genes were identified using the RNA-seq analysis in *Miichthys miiuy* (Che et al., 2014), *O. mykiss* (Ali et al., 2014) and *Schizothorax prenanti* (Luo et al., 2016). It is generally thought that stress depresses immune functioning in humans (Herbert & Cohen, 1993). This connection between stress and immune functions is also apparent in vertebrates (Tort, 2011). High-throughput sequencing could help identify the immune related genes in chum salmon. “Salmonella infection” and “herpes simplex infection” were found in the immune response cluster, which could indicate the chum salmon were infected with Salmonella and Herpes simplex in higher salinity environments. There is a possibility that chum salmon carry out a series of anti-inflammatory activities by stimulating the immune system in different salinity environments. For example, to maintain normal bodily functions, a large number of non-specific immune enzymes are produced to remove oxygen free radicals and catalyze the hydrolysis of phosphorus containing compounds to remove metabolic waste in the body. The annotated unigenes could participate in various biological processes that help explain this result. Transcriptome studies have also been done in *Larimichthys crocea*, *Gymnocypris przewalskii*, and *Megalobrama amblycephala* (Xiao et al., 2015a; Tran et al., 2015; Tong et al., 2015). These salinity-regulated unigenes are associated with cellular processes, environmental alteration, genetic information, the immune system (immune response in humans), metabolism and organismal systems for salinity adaptation.

### Effects of salinity on growth-related and memory-related genes in chum salmon

The growth-related genes *igf-1* and *igfbp* were previously investigated in salmon under salinity changes (Shepherd et al., 2005; Sharif et al., 2015). One study found that the muscle *igf-1* of chum salmon has no effect on seawater transfer processing (Taniyama et al., 2016), and that gene *igf-1* in vertebrates is stimulated in the somatic and skeletal muscle growth process (Wood, Duan & Bern, 2005). Gene *igfbp-1* of juvenile chum salmon liver was negatively correlated with growth rate in previous studies (Hiroyasu et al., 2008; Pedroso, Fukada & Masumoto, 2009; Peterson & Waldbieser, 2009; Kawaguchi et al., 2013; Breves et al., 2014). Muscle *igfbp-1a* and *igfbp-1b* responded to fasting, but the *igfbp-1b* levels were much lower. Larger zebrafish showed a lower muscle expression of *igfbp-1a* and *igfbp-1b* compared to smaller zebrafish (Amaral & Johnston, 2012).

Memory-related *nmda* gene expression is more strongly altered with salinity changes. The increases in *nmdas* may be due to increased dopamine secretion under the salinity changes. The increase of *nmda1* expression in chum salmon could change the learning and memory capacities of the fish during the transition from freshwater to seawater (Yu et al., 2014). Dopamine affects learning ability, the formation of olfactory glomeruli and long-term storage memory (Hsia, Vincent & Lledo, 1999; Pignatelli et al., 2005). Dopamine has also been found to influence the migration of cells in European eel (*Anguilla anguilla*) and chum salmon (Finn-Arne et al., 2006; Kim et al., 2015). In this study, the relative expression of growth-related genes and memory-related genes was lowest in the D8 and D16 groups, and highest in the D24 group. These genes may not exhibit higher expression in the D8 and D16 groups as the D8 and D16 salinity conditions may represent a more suitable environment for juvenile chum salmon than higher levels of salinity.

### SNPs of chum salmon

SNPs are widely used in genome studies (Xiao et al., 2016), the construction of genetic maps, and the analysis of population genetics in the transcriptomes of organisms (Xiao et al., 2015b; Xia et al., 2014). The chum salmon genome has not yet been released as there are not sufficient SNP markers for genetic analysis in chum salmon. In previous studies, nearly 26,000 putative SNPs were identified in individual chum salmon (Seeb et al., 2011). However, salinity-related SNPs of chum salmon were rarely reported in previous studies. In this study, 32,468 SNPs in the D0 group and 36,289 SNPs in the D24 group were found with the AG/GA and CT/TC types the most abundant, and the GC/CG types the least abundant. This variation might cause base structure differences in the DNA sequence of chum salmon (Ma et al., 2012). A total of 56,020 SNP positions differed between the two groups in response to salinity changes. The gene structure of the small variants may be for the chum salmon to survive the migration from freshwater to saltwater. These SNPs may provide abundant molecular resources on quantitative trait locus (QTL) studies and molecular marker-assisted selection for population genetic structures in chum salmon. The SNP markers were designed for validating and testing chum salmon populations in future studies.

## Conclusion

In this study, the ATPase and antioxidant enzymes, gene expression, and SNPs associated with salinity adaptation in chum salmon were studied using a comparative transcriptome analysis. The ATPase and antioxidant enzymes varied in the different salinity environments. A total of 2,143 differentially expressed genes were identified in RNA-seq, and 56,020 SNP positions differed between the D0 and the D24 groups. These results could provide valuable genetic resources and molecular marker-assisted breeding opportunities for chum salmon.

## Supplemental Information

10.7717/peerj.13585/supp-1 Supplemental Information 1 Raw data for Figures 1, 2 and 6.

10.7717/peerj.13585/supp-2 Supplemental Information 2 Length distribution of assembled unigenes in the chum salmon transcriptome.

10.7717/peerj.13585/supp-3 Supplemental Information 3 A power analysis calculation for the chum salmon transcriptome in D0 and D24 group.

10.7717/peerj.13585/supp-4 Supplemental Information 4 The read depth for the chum salmon transcriptome in D0 and D24 groups.

10.7717/peerj.13585/supp-5 Supplemental Information 5 The co-expressed genes of the chum salmon transcriptome in D0 and D24 groups.

10.7717/peerj.13585/supp-6 Supplemental Information 6 The differential genes expression in KEGG pathways. a. the up-regulated genes; b. the down-regulated genes.

10.7717/peerj.13585/supp-7 Supplemental Information 7 The cleaned data of chum salmon transcriptome.

10.7717/peerj.13585/supp-8 Supplemental Information 8 DEGs related to salinity.

10.7717/peerj.13585/supp-9 Supplemental Information 9 SNP numbers.

10.7717/peerj.13585/supp-10 Supplemental Information 10 DEGs annotation.

10.7717/peerj.13585/supp-11 Supplemental Information 11 SNPs.

10.7717/peerj.13585/supp-12 Supplemental Information 12 Unigenes.

10.7717/peerj.13585/supp-13 Supplemental Information 13 Unigene annotation.

10.7717/peerj.13585/supp-14 Supplemental Information 14 Author Checklist.

## Additional Information and Declarations

### DNA Deposition

The following information was supplied regarding the deposition of DNA sequences: The raw data is available at NCBI: PRJNA778360 and ON804215-ON804220

### Data Availability

The following information was supplied regarding data availability: The unigenes, the unigenes annotation, DEG annotation, SNPs, raw data of Figures 1, 2 and 6 are available in the Supplemental File.
