# Supplementary figures and images for: Biochemical indices, gene expression, and SNPs associated with salinity adaptation in juvenile chum salmon (Oncorhynchus keta) as determined by comparative transcriptome analysis
Source: PeerJ. 2022 Sep 12;10:e13585. doi: 10.7717/peerj.13585 (PMC9477081; doi:10.7717/peerj.13585)

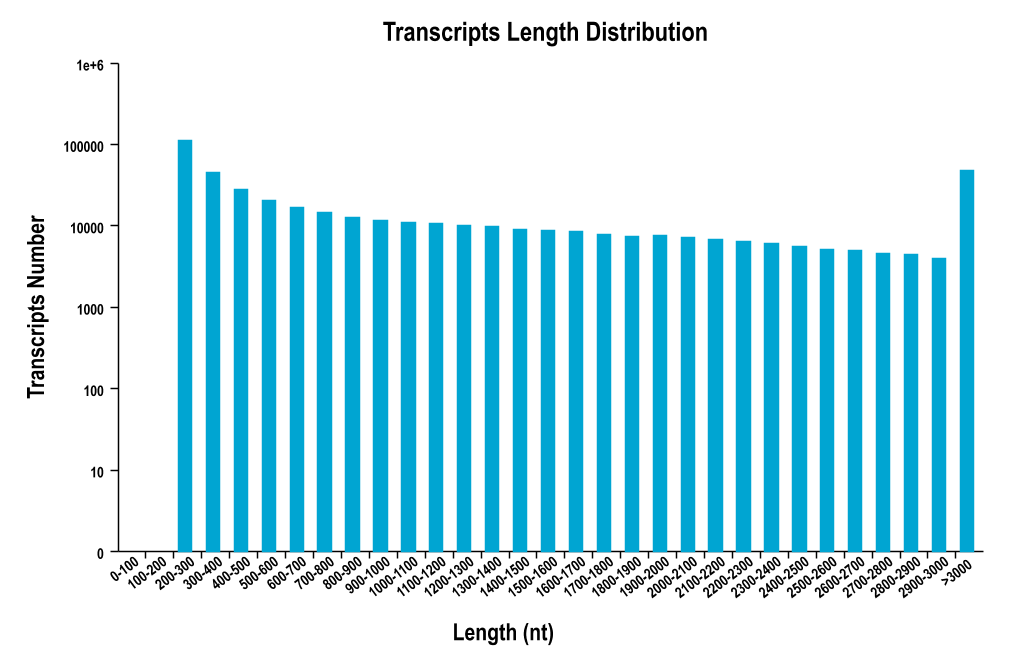

Supplement: Supplemental Information 2 [file peerj-10-13585-s002.png]

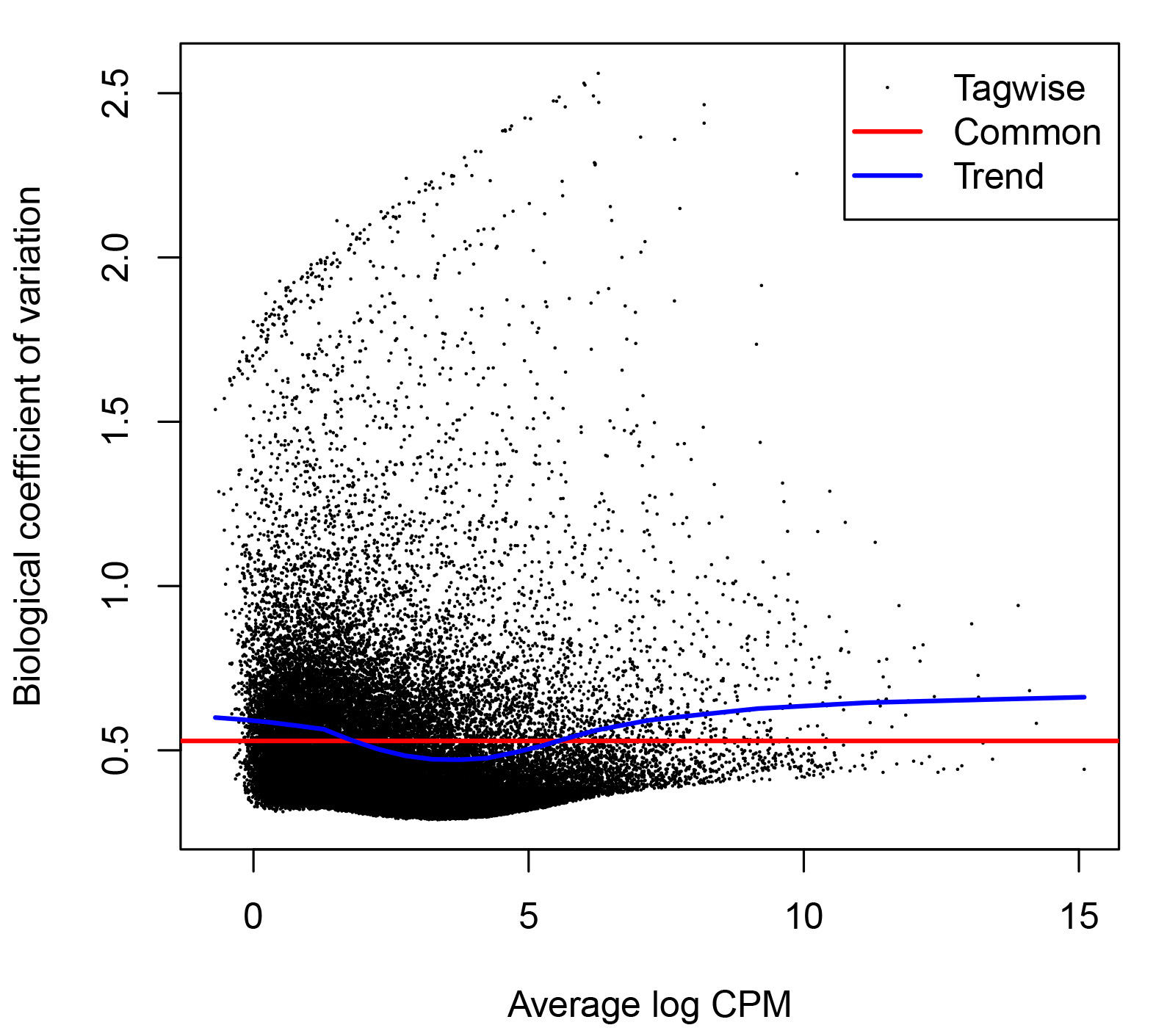

Supplement: Supplemental Information 3 [file peerj-10-13585-s003.png]

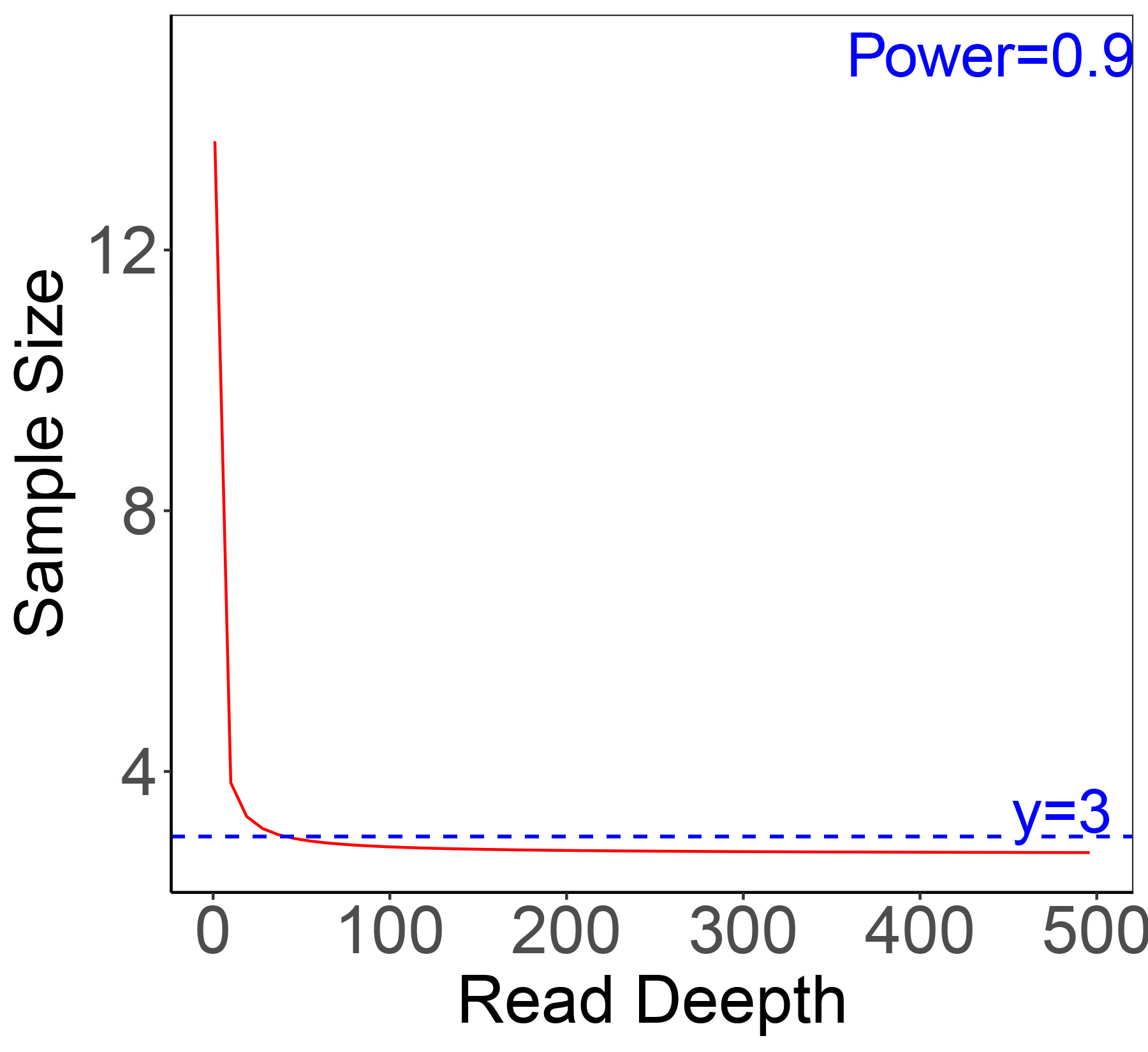

Supplement: Supplemental Information 4 [file peerj-10-13585-s004.png]

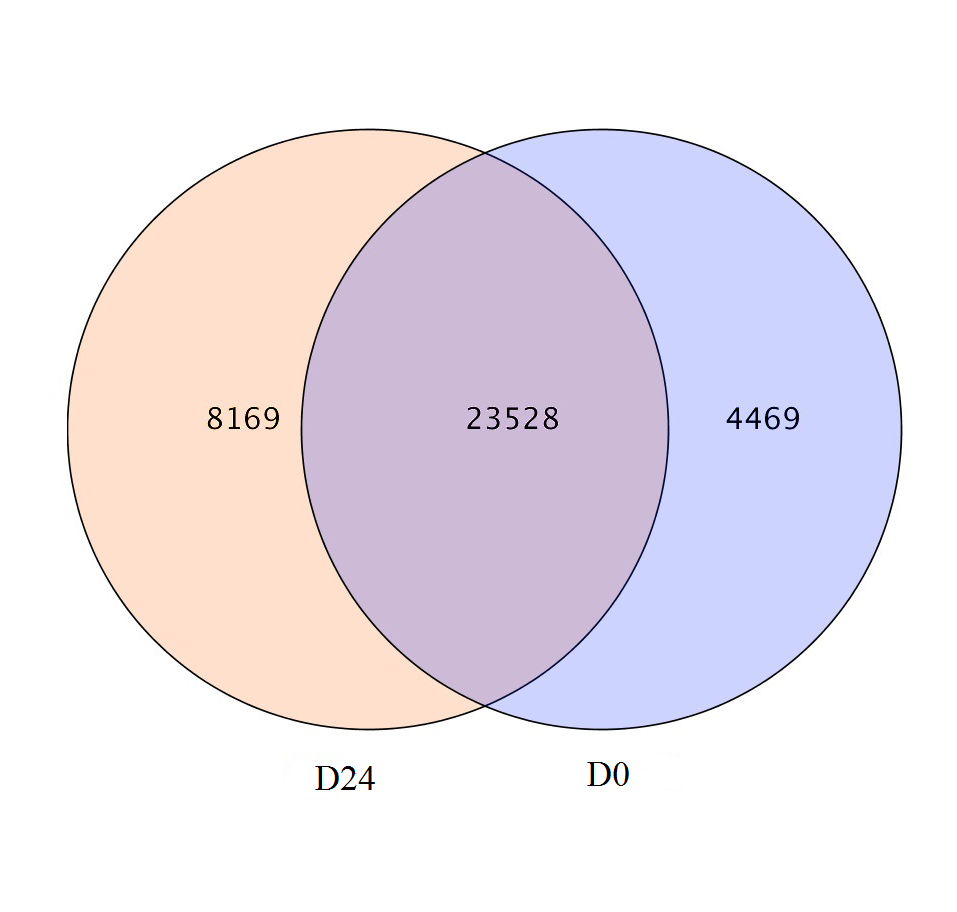

Supplement: Supplemental Information 5 [file peerj-10-13585-s005.png]

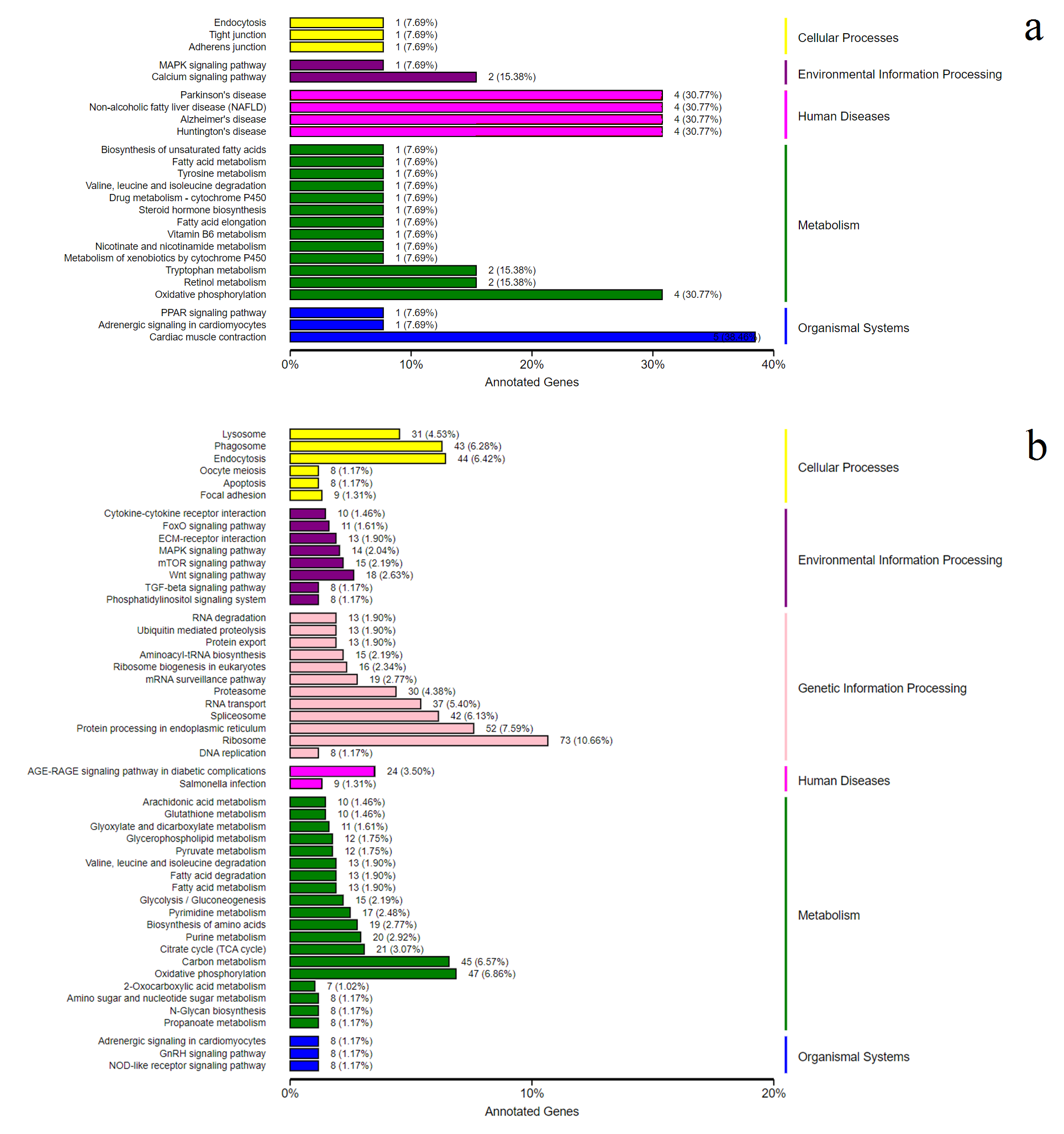

Supplement: Supplemental Information 6 — a. the up-regulated genes; b. the down-regulated genes. [file peerj-10-13585-s006.png]
