# Supplementary material for: Biochemical indices, gene expression, and SNPs associated with salinity adaptation in juvenile chum salmon (Oncorhynchus keta) as determined by comparative transcriptome analysis
Source: PeerJ. 2022 Sep 12;10:e13585. doi: 10.7717/peerj.13585 (PMC9477081; doi:10.7717/peerj.13585)
Supplement: Supplemental Information 7 [file peerj-10-13585-s007.docx]

Table S1 The cleaned data of chum salmon transcriptome.

| Group | Clean Reads | Mapped Reads | Mapped Ratio |
| --- | --- | --- | --- |
| D0F1 | 28065353 | 17168282 | 61.17% |
| D0F2 | 23350030 | 14593357 | 62.50% |
| D0F3 | 29186035 | 19021277 | 65.17% |
| D24F1 | 27406560 | 16250978 | 59.30% |
| D24F2 | 29214624 | 17951025 | 61.45% |
| D24F3 | 33195313 | 20359813 | 61.33% |
