# Supplementary material for: Biochemical indices, gene expression, and SNPs associated with salinity adaptation in juvenile chum salmon (Oncorhynchus keta) as determined by comparative transcriptome analysis
Source: PeerJ. 2022 Sep 12;10:e13585. doi: 10.7717/peerj.13585 (PMC9477081; doi:10.7717/peerj.13585)
Supplement: Supplemental Information 8 [file peerj-10-13585-s008.docx]

Table S2 DEGs related to sanility.

| Go. ID | Term | Annotated unigenes number |
| --- | --- | --- |
| GO:0042538 | hyperosmotic salinity response | BMK_Unigene_000119;BMK_Unigene_000121;BMK_Unigene_000123;BMK_Unigene_004927;BMK_Unigene_007499;BMK_Unigene_011424;BMK_Unigene_015186;BMK_Unigene_016214;BMK_Unigene_018937;BMK_Unigene_027489;BMK_Unigene_047112;BMK_Unigene_055003;BMK_Unigene_075388;BMK_Unigene_075904;BMK_Unigene_088301;BMK_Unigene_141495;BMK_Unigene_144987;BMK_Unigene_157198;BMK_Unigene_161742;BMK_Unigene_172780;BMK_Unigene_200248;BMK_Unigene_224653;BMK_Unigene_234107;BMK_Unigene_246961;BMK_Unigene_254487;BMK_Unigene_254627;BMK_Unigene_327521;BMK_Unigene_335828;BMK_Unigene_345151;BMK_Unigene_348043;BMK_Unigene_360035;BMK_Unigene_375126 |
| GO:0071477 | cellular hypotonic salinity response | BMK_Unigene_020335;BMK_Unigene_022432;BMK_Unigene_042523;BMK_Unigene_160502;BMK_Unigene_253844;BMK_Unigene_256640 |
| GO:0042539 | hypotonic salinity response | BMK_Unigene_006492;BMK_Unigene_020335;BMK_Unigene_022432;BMK_Unigene_042523;BMK_Unigene_058480;BMK_Unigene_059112;BMK_Unigene_069294;BMK_Unigene_088301;BMK_Unigene_145802;BMK_Unigene_160502;BMK_Unigene_206336;BMK_Unigene_241534;BMK_Unigene_253393;BMK_Unigene_253844;BMK_Unigene_256640;BMK_Unigene_357043;BMK_Unigene_365089;BMK_Unigene_367731 |
