# Supplementary material for: Biochemical indices, gene expression, and SNPs associated with salinity adaptation in juvenile chum salmon (Oncorhynchus keta) as determined by comparative transcriptome analysis
Source: PeerJ. 2022 Sep 12;10:e13585. doi: 10.7717/peerj.13585 (PMC9477081; doi:10.7717/peerj.13585)
Supplement: Supplemental Information 9 [file peerj-10-13585-s009.docx]

Table S3 SNPs numbers.

| Group | HomoSNP | HeteSNP | AllSNP |
| --- | --- | --- | --- |
| D0F1 | 429063 | 61233 | 490296 |
| D0F2 | 373146 | 57558 | 430704 |
| D0F3 | 463733 | 75975 | 539708 |
| D24F1 | 437799 | 59882 | 497681 |
| D24F2 | 462014 | 65648 | 527662 |
| D24F3 | 474696 | 67065 | 541761 |
